# Trifluoperazine reduces apoptosis and inflammatory responses in traumatic brain injury by preventing the accumulation of Aquaporin4 on the surface of brain cells

**DOI:** 10.7150/ijms.82677

**Published:** 2023-04-29

**Authors:** Rongchun Xing, Jin Cheng, Jiangtao Yu, Shaoping Li, Haoli Ma, Yan Zhao

**Affiliations:** 1 Emergency Center, Zhongnan Hospital of Wuhan University, Wuhan 430071, China.; 2 Department of Biological Repositories, Zhongnan Hospital of Wuhan University, Wuhan 430071, China.; 3 Hubei Clinical Research Center for Emergency and Resuscitation, Zhongnan Hospital of Wuhan University, Wuhan 430071, China.

**Keywords:** traumatic brain injury, rat cortex, trifluoperazine, AQP4, apoptosis, inflammation

## Abstract

Currently, no specific and standard treatment for traumatic brain injury (TBI) has been developed. Therefore, studies on new therapeutic drugs for TBI treatment are urgently needed. Trifluoperazine (TFP) is a therapeutic agent for the treatment of psychiatric disorders that reduces edema of the central nervous system. However, the specific working mechanism of TFP is not fully understood in TBI. In this study, the immunofluorescence co-localization analysis revealed that the area and intensity covered by Aquaporin4 (AQP4) on the surface of brain cells (astrocyte endfeet) increased significantly after TBI. In contrast, TFP treatment reversed these phenomena. This finding showed that TFP inhibited AQP4 accumulation on the surface of brain cells (astrocyte endfeet). The tunel fluorescence intensity and fluorescence area were lower in the TBI+TFP group compared to the TBI group. Additionally, the brain edema, brain defect area, and modified neurological severity score (mNSS) were lower in the TBI+TFP. The RNA-seq was performed on the cortical tissues of rats in the Sham, TBI, and TBI+TFP groups. A total of 3774 genes differently expressed between the TBI and the Sham group were identified. Of these, 2940 genes were up-regulated and 834 genes were down-regulated. A total of 1845 differently expressed genes between the TBI+TFP and TBI group were also identified, in which 621 genes were up-regulated and 1224 genes were down-regulated. Analysis of the common differential genes in the three groups showed that TFP could reverse the expression of apoptosis and inflammation genes. Gene ontology (GO) and Kyoto Encyclopedia of Genes and Genomes (KEGG) enrichment analysis revealed that the differentially expressed genes (DEGs) were highly enriched in the signaling pathways regulating inflammation. In conclusion, TFP alleviates brain edema after TBI by preventing the accumulation of AQP4 on the surface of brain cells. Generally, TFP alleviates apoptosis and inflammatory response induced by TBI, and promotes the recovery of nerve function in rats after TBI. Thus, TFP is a potential therapeutic agent for TBI treatment.

## 1. Introduction

Traumatic brain injury (TBI) is caused by external forces to the head, and mainly causes brain dysfunction 1. Statistics show that more than 50 million people worldwide suffer traumatic brain injury annually, and about half of the world's population suffer at least one traumatic brain injury in their lifetime 2. TBI is one of the leading causes of death among young and middle-aged people, and the leading cause of disability or death at all ages 2. TBI causes approximately $400 billion losses yearly 2. The global burden of TBI has been increasing significantly 3. The incidence of TBI in poor populations is particularly high 4, 5. Overall, TBI exerts a significant economic burden to society 2.

Traumatic brain injury causes acute lesions in the central nervous system. The pathophysiological process of TBI is complex and dynamic, and the disease is refractory 6. The pathophysiological mechanism of TBI includes two aspects: primary injury caused by direct mechanical force and secondary injury of the neurons. The mechanical force caused by the impact can directly damages the axon and causes partial neural damage 7. Secondary brain injury occurs through several pathophysiological processes, including electrolyte imbalance 8, mitochondrial dysfunction 9, neuroinflammation 10, cerebral edema 11, and cerebrovascular injury 12. Although several mechanisms have been studied, the regulation mechanism between various genes and signaling pathways has not been fully elucidated. TBI treatments include surgery and medications such as statins, progesterone, cyclosporin, erythropoietin, and tranexamic acid 6. However, Clinical trials of most drugs have shown no particular effect 6. Currently, the treatment focuses on acute symptomatic management rather than targeting specific pathophysiological processes or signaling pathway targets after TBI 13. Therefore, it is particularly important to understand the possible causes influencing the efficacy of TBI drug therapy and explore the molecular and complex pathway changes before and after TBI treatment.

Cerebral edema is a common pathological condition caused by TBI. The severity of cerebral edema and whether the edema can subside as soon as possible are closely related to the prognosis. Researchers have been developing specific drugs to inhibit the progression of cerebral edema. However, all studies have failed in phase III of the clinical trials. Currently, symptomatic treatment remains the main approach for TBI treatment. The symptomatic treatment focuses on reducing the edema state rather than the cause of the edema.

AQP4 is expressed in astrocyte cell membrane and is most abundant in its endfoot, AQP4 in astrocyte endfeet are mainly located on the surface of capillaries 14. AQP4 is an important water transporter. Studies have found that AQP4 plays an important role in the occurrence and development of cerebral edema.The expression of AQP4 in the brain is elevated after TBI 15. Intervention of AQP4 expression on the surface of brain cells may be an effective method to treat cerebral edema after TBI. Trifluoperazine (TFP) is a calmodulin inhibitor that has been approved as an antipsychotic by the US Food and Drug Administration and the UK's National Institute for Health and Care Excellence 16. In recent years, it has been found that TFP can affect the subcellular localization of AQP4 in brain cells to relieve central nervous system edema 16. However, the gene regulation mechanism involved in the TFP treatment of brain edema and the complex signal pathway is yet to be elucidated. In the present study, a rat model revealed that TFP alleviates brain edema and improves neural function after TBI. Meanwhile, the genes and signaling pathways targeted by TFP were identified using whole genome analysis of RNA-seq. It further provided theoretical support for the treatment of TBI by TFP.

## 2. Materials and Methods

### 2.1 Animals

A total of 66 male Sprague-Dawley rats (8 weeks, weighing 280-330g) were purchased from Vital River Experimental Animal Technology Co., LTD. (Vital River, Beijing, China). At room temperature every 12-hour light/dark cycle, free access to food and water. The rats were fed for one week in the Animal Experimental Center of Zhongnan Hospital of Wuhan University before surgery. The study protocols were approved by the Animal Laboratory Center and Ethics Committee of Zhongnan Hospital of Wuhan University, and the animal experiments were conducted in accordance with the Guidelines of the National Institutes of Health for the Care and Application of Laboratory Animals in China.

### 2.2 Traumatic brain injury model

The rats were randomly divided into the Sham (22 rats) and brain trauma (44 rats) groups. Cerebral cortex contusion was induced using an impact weight down device as previously described 17, 18. Briefly, the rats were injected intraperitoneally with 1% pentobarbital anesthesia (40 mg/kg). A scalp midline incision was then made under aseptic conditions. After exposing the skull, a hole about 5 mm wide was drilled on the right side, exposing the dura mater. A 50 g weight was dropped vertically from a height of 25 cm, hitting the exposed brain tissue, causing moderate traumatic brain injury. The rats in the Sham group were craniotomized in a similar manner, but the brain tissue was not damaged. After 72 hours, 42 rats in the sham and the brain trauma groups were sacrificed, and brain tissues were extracted for pathological and brain edema analysis, and RNA sequencing. All the brain tissues for sequencing were stored in the refrigerator at -80°C. The remaining 24 rats were sacrificed by intraperitoneal injection of 1% pentobarbital (200 mg/kg) after behavioral test 1 week later.

### 2.3 Trifluoperazine treatment and experimental group

The rats were randomly divided into three groups: Sham+saline group, TBI+saline group and TBI+TFP group, which were represented by Sham, TBI and TBI+TFP in the following sections. Each group comprised 15 rats. Trifluoperazine (TFP) (Blue Wood Chemical Co. LTD, Shanghai, China) was dissolved in normal saline. The TFP concentration for the TBI+TFP group was 5 mg/kg. Intraperitoneal injection was performed one hour after TBI. The Sham and TBI groups received intraperitoneal injections of equal volumes of normal saline. The injection dose, treatment window, and administration mode were based on preliminary experimental results and references to previous studies 19, 20.

### 2.4 Immunofluorescence test

The rats (n=3 in each group) were anesthetized and their hearts were exposed at 72h after TBI. Briefly, prior to harvesting the rat brain tissue, the heart and blood vessels were rinsed with saline (50 ml) and then 4% paraformaldehyde (100 mL) was infused through the heart into the brain tissue. The brain was removed and placed in 4% paraformaldehyde for 24 hours. After embedding in paraffin, the brain tissues were cut into thin sections covering the damaged area. The brain tissues were incubated at 65℃ for 2h, dewaxed, and washed three times with 0.01 MPBS for 5 min each time. Antigen was extracted with EDTA repair solution and washed three times with PBS. The samples were incubated in the dark with 3% hydrogen peroxide (Biosharp, Beijing, China) for 10 min, and thereafter with anti-CD31 (ABclone, 1:100) (cerebrovascular endothelial cells, red), GFAP (ABclone, 1:100) (astrocytes, red), IBA1 (ABclone, 1:100) (microglia, Red), and AQP4 (ABclone, 1:100) (green) primary antibodies at 4℃ overnight (15 h). After rinsing with PBS, the brain tissues were incubated for 1 h with the secondary antibody. Finally, the samples were incubated with DAPI, covered with a cover slip and observed under a fluorescence microscope (Olympus, BX53). In the immunofluorescence experiment, three samples were taken from each group, and six different areas were selected from brain tissue slices of rats in each group to calculate the area and intensity of immunofluorescence. Image J (v8.0.1, SanDiego, USA) software was used to calculate the area and the intensity of immunofluorescence.

Apoptotic cells on paraffin-embedded brain tissue sections were detected by an in-situ apoptosis detection kit (Boster, Wuhan, China). Three samples were taken from each group for this experiment, observed under a fluorescence microscope (Olympus, BX53), and six different areas were selected from the brain tissue of rats in each group to calculate the immunofluorescence area and intensity based on tunel positive cells. Image J (v8.0.1, SanDiego, USA) software was used to calculate the area and the intensity of immunofluorescence.

### 2.5 HE staining and lesion area assessment

At 72h after TBI, 3 complete brain tissues were taken from each group after anesthesia. The paraffin embedding and sectioning methods were similar to the previous immunofluorescence steps. The brain sections were stained with HE stain (Solarbio, Beijing, China), observed under a fluorescence microscope (Olympus, BX53). The brain defect area was calculated using the Image J (v8.0.1, SanDiego, USA) software. Tissue area loss was calculated by subtracting the ipsilateral area (damaged hemisphere) from the contralateral area (control hemisphere) 21.

### 2.6 Determination of cerebral water content

At 72 hours after TBI, the rats were killed after anesthesia, and the brain tissue was taken. The brain water content levels were calculated using the wet and dry weight method 22. A sample from the injured cerebral cortex was obtained within a 5mm diameter. A similar measurement for the control sample was obtained from the contralateral cortex. The wet weight of each sample was immediately weighed and dried in a drying oven at 65℃ for 24 hours to obtain the dry weight. The percentage of water content was calculated as water content (%) = [(wet weight-dry weight)/wet weight] x100% 22.

### 2.7 Modified neurological function severity score (mNSS)

Modified neurological function severity score (mNSS) was measured at 1, 3, 5, and 7 days after TBI to assess the neurological function (n=8 per group). The mNSS consists of motor, sensory, balance, and reflex tests. The highest scores on the four tests were 6, 2, 6, and 4. The higher the score, the higher the severity of the neurological defects 23.

### 2.8 Analysis of RNA omics data

The cerebral cortex tissues for rat in the Sham, TBI, and TBI+TFP were extracted for RNA sequence analysis. Briefly, total RNA was extracted using TRIzol reagents (Invitrogen, Carlsbad, CA, USA), according to the manufacturer's instructions. RNA purity and integrity were assessed using the NanoDrop (Thermo Fisher, USA) and Bioanalyzer 2100 systems (Agilent Technologies, USA). Library construction was performed with NEB Next Ultra RNA Library Prep Kit for Illumina (NEB, USA). Agilent 2100 bioanalyzer and Q-PCR (effective concentration >2 nM) were used to evaluate the quality and effective concentration of the sequencing library. Raw reads were first processed through Trimmomatic (v0.30) to obtain high-quality clean data (clean reads). The reference Genome and gene model annotation files were downloaded directly from the Genome website. Reference genome alignment was performed using Hisat2 (v2.0.1). Based on the Hisat2 alignment results, stringtie was used to reconstruct transcripts and the expression levels of all genes in each sample were calculated. The mRNA expression level was calculated based on the FPKM value (Fragments Per Kilobase Million). The differentially expressed mRNA were identified based on |log2 (a fold change)|> 1 and P-value < 0.05, DEGSeq was used for transcriptome differential expression analysis. The DEGs were displayed using Heat maps and Venn diagrams. The GO and KEGG enrichment analysis of the DEGs were also performed.

### 2.9 Statistical Analysis

Data were analyzed using Prism software (v8.0.1, SanDiego, USA). Except for transcriptomic data, other data were expressed as mean ± standard deviation (SD). Student's t-test was used for data analysis. P-value < 0.05 was considered statistically significant.

## 3. Results

### 3.1 Immunofluorescence and apoptosis

**Figure 1:**
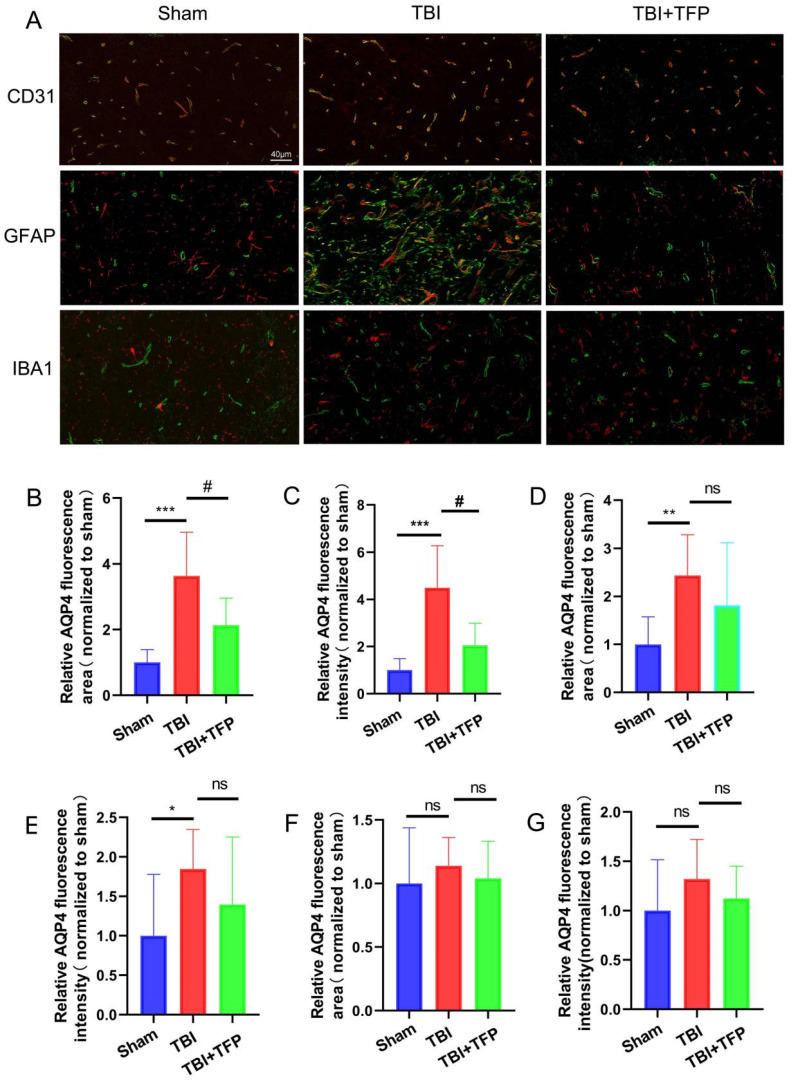
The expression of AQP4 around brain cells assessed using the immunofluorescence co-localization method. A: The expression of AQP4 (green) on cerebrovascular endothelial cells (CD31, red), astrocytes (GFAP, red), and microglia (IBA1, red) in each group (Sham, TBI, and TBI+TFP groups); B and C: The relative fluorescence area (TBI vs Sham, ***P<0.001; TBI+TFP vs TBI, ^#^P<0.05) and the relative fluorescence intensity (TBI vs Sham, ***P<0.001; TBI+TFP vs TBI, ^#^P<0.05) of AQP4 around cerebral vascular endothelial cells in each group (Sham, TBI, and TBI+TFP groups); D and E: The relative fluorescence area (TBI vs Sham, **P<0.01; TBI+TFP vs TBI, P>0.05) and the relative fluorescence intensity (TBI vs Sham, *P<0.05; TBI+TFP vs TBI, P>0.05) of AQP4 around astrocytes of each group (Sham, TBI, and TBI+TFP groups); F and G: The relative fluorescence area (TBI vs Sham, P>0.05; TBI+TFP vs TBI, P>0.05) and the relative fluorescence intensity (TBI vs Sham, P>0.05; TBI+TFP vs TBI, P>0.05) of AQP4 around microglia of each group (Sham, TBI, and TBI+TFP groups). The results of Figures 1B, 1C, 1D, 1E, 1F, and 1G were obtained by Sham standardization (n=3 for each group above).

Immunofluorescence co-localization method was used to detect the expression of AQP4 around cerebrovascular endothelial cells (CD31), astrocytes (GFAP) and microglia (IBA1) in each group (Sham group, TBI group, TBI+TFP group) (Figure 1A). (1) Compared with the Sham group, the relative fluorescence area (TBI vs Sham, P=0.0009) and fluorescence intensity (TBI vs Sham, P=0.0009) of AQP4 around cerebral vascular endothelial cells (CD31, red) increased after TBI modeling, while the relative fluorescence area (TBI+TFP vs TBI, p=0.0404) and fluorescence intensity (TBI+TFP vs TBI, P=0.0138) of AQP4 around cerebral vascular endothelial cells decreased after TFP treatment, as shown in Figures 1B and 1C. (2) Compared with the Sham group, the relative fluorescence area (TBI vs Sham, P=0.0063) and fluorescence intensity (TBI vs Sham, P=0.0490) of AQP4 around brain astrocytes in the TBI group increased, while there was no difference in the statistical results of the relative fluorescence area (TBI+TFP vs TBI, P=0.3480) and fluorescence intensity (TBI+TFP vs TBI, P=0.2924) of AQP4 around cerebral vascular endothelial cells after TFP treatment, as shown in Figures 1D and 1E. (3) Compared with Sham group, there were no difference in the relative fluorescence area (TBI+TFP vs TBI, P=0.5011) and fluorescence intensity (TBI+TFP vs TBI, P=0.2578) of AQP4 around brain microglia in TBI group. However, there was no difference in the relative fluorescence area (TBI+TFP vs TBI, P=0.5219) and fluorescence intensity (TBI+TFP vs TBI, P=0.3756) of AQP4 around brain microglia cells after TFP treatment, as shown in Figures 1F and 1G. (n=3 in each groups). Fluorescence area and amount were calculated by Image J (v8.0.1, SanDiego, USA). Statistical results of the above figures were obtained after standardization of Sham group.

**Figure 2:**
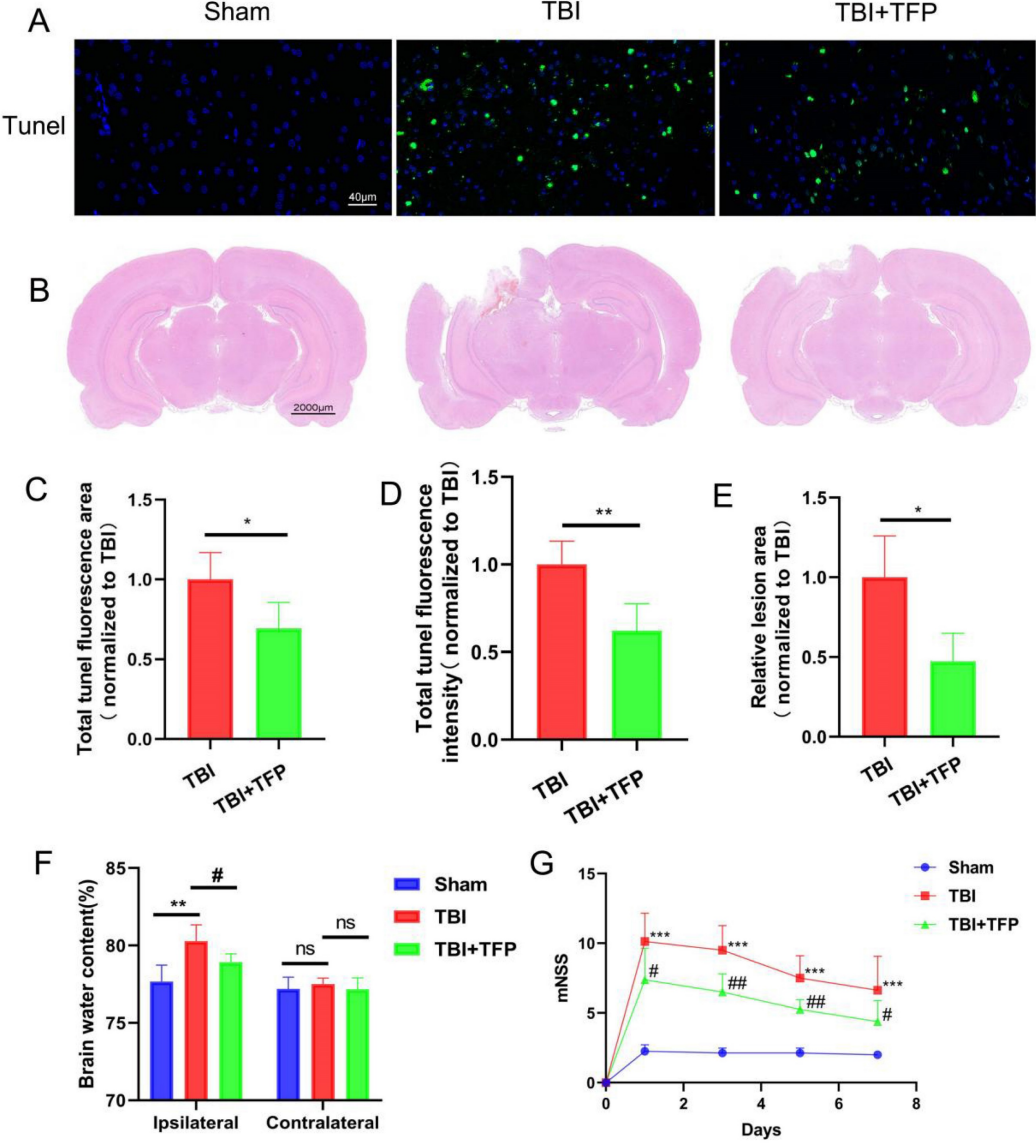
Comparison of neuronal apoptosis, lesion area of brain tissue, and behavioral indexes in each group (Sham, TBI, and TBI+TFP groups). A: The expression of tunel signal (green) in brain tissue in the Sham, TBI, and TBI+TFP groups; B: Brain lesion area of each group (Sham, TBI, and TBI+TFP groups) after HE staining; C and D: Quantified comparison charts of tunel fluorescence area (TBI+TFP vs TBI, *P<0.05) and fluorescence intensity (TBI+TFP vs TBI,**P<0.05) in the Sham, TBI, and TBI+TFP vs TBI groups, (n=3); E: A quantified comparison chart of brain lesion area between TBI and TBI+ TBI groups (TBI+TFP vs TBI, *P<0.05) (n=3 in each group); F: The dry-wet ratio of water content on the side of brain injury in each group (Sham, TBI, and TBI+TFP groups) (TBI vs Sham, **P<0.01; TBI+TFP vs TBI, ^#^P<0.05) and the dry-wet ratio of cerebral water content in contralateral cortex (TBI vs Sham, P>0.05; TBI+TFP vs TBI, P>0.05) (n=5); G: A comparison of mNSS behavioral scores in the Sham, TBI, and TBI+TFP groups. The mNSS scores were performed at 1, 3, 5, and 7 days after TBI. The mNSS scores in the TBI group were significantly higher than those in the Sham group (***P-value in all groups was less than 0.001). Compared with the TBI group, the scores in the TBI+TFP group on days 1, 3, 5, and 7 decreased (^#^P<0.05, ^##^P<0.01, ^##^P<0.01, ^#^P<0.05) (n=8). The results of Figures 2B, 2C, and 2E were obtained by Sham standardization.

Apoptosis kit was used to detect neuronal apoptosis of brain cells (Figure 2A). After TFP treatment, the total fluorescence area (TBI+TFP vs TBI, P=0.0097) and fluorescence intensity (TBI+TFP vs TBI, P=0.0011) of tunel (green) in brain cells were decreased compared with that in TBI group (Figures 2C and 2D), indicating that TFP reduces apoptosis of brain cells (n=3 in each group). fluorescence area and fluorescence intensity were calculated by Image J (v8.0.1, SanDiego, USA). All calculation results in these figures are obtained after standardization by the TBI group.

### 3.2 HE staining and lesion area results

To evaluate the effect of TFP on pathological defect area of brain tissue after TBI (Figure 2B). HE staining showed that compared with the TBI group, the lesion area of brain injury was significantly lower in the TBI+TFP group (TBI+TFP vs TBI, P=0.0433) (n=3 in each group) (Figure 2E).

### 3.3 Cerebral water content

Brain water content was calculated by the wet-dry ratio. We observed significant differences in cerebral water content in the ipsilateral hemisphere of rats between the TBI and sham group (80.28±1.05 vs 77.67±1.07, P=0.0046) and between the TBI+TFP and the TBI group (78.92±0.54 vs 80.28±1.05, P=0.0336). However, no significant differences were observed in the in cerebral water content in the contralateral hemisphere among the Sham, TBI, and TBI+TFP groups (TBI vs Sham, 77.51±0.38 vs 77.20±0.76, P=0.4353; TBI+TFP vs TBI, 77.20±0.76 vs 77.17±0.74, P=0.3893), (n=5 in each group) (Figure 2F).

### 3.4 TFP alleviated neural damage

The Modified Neurological Severity Score (mNSS) was used to evaluate the effect of TFP on improving neurological function after TBI (Figure 2G). The nerve damage was assessed on days 1, 3, 5, and 7 after TBI. The mNSS scores were significantly higher in the TBI group than in the Sham group (P-value <0.001 in all groups). Compared with the TBI group and based on the mNSS scores, the severity of nerve damage was lower in the TBI+TFP group on days 1, 3, 5, and 7 decreased (P=0.0228, p=0.0018, P=0.0027, P=0.0438) (n=8 in each group).

### 3.5 RNA sequencing analysis

**Figure 3:**
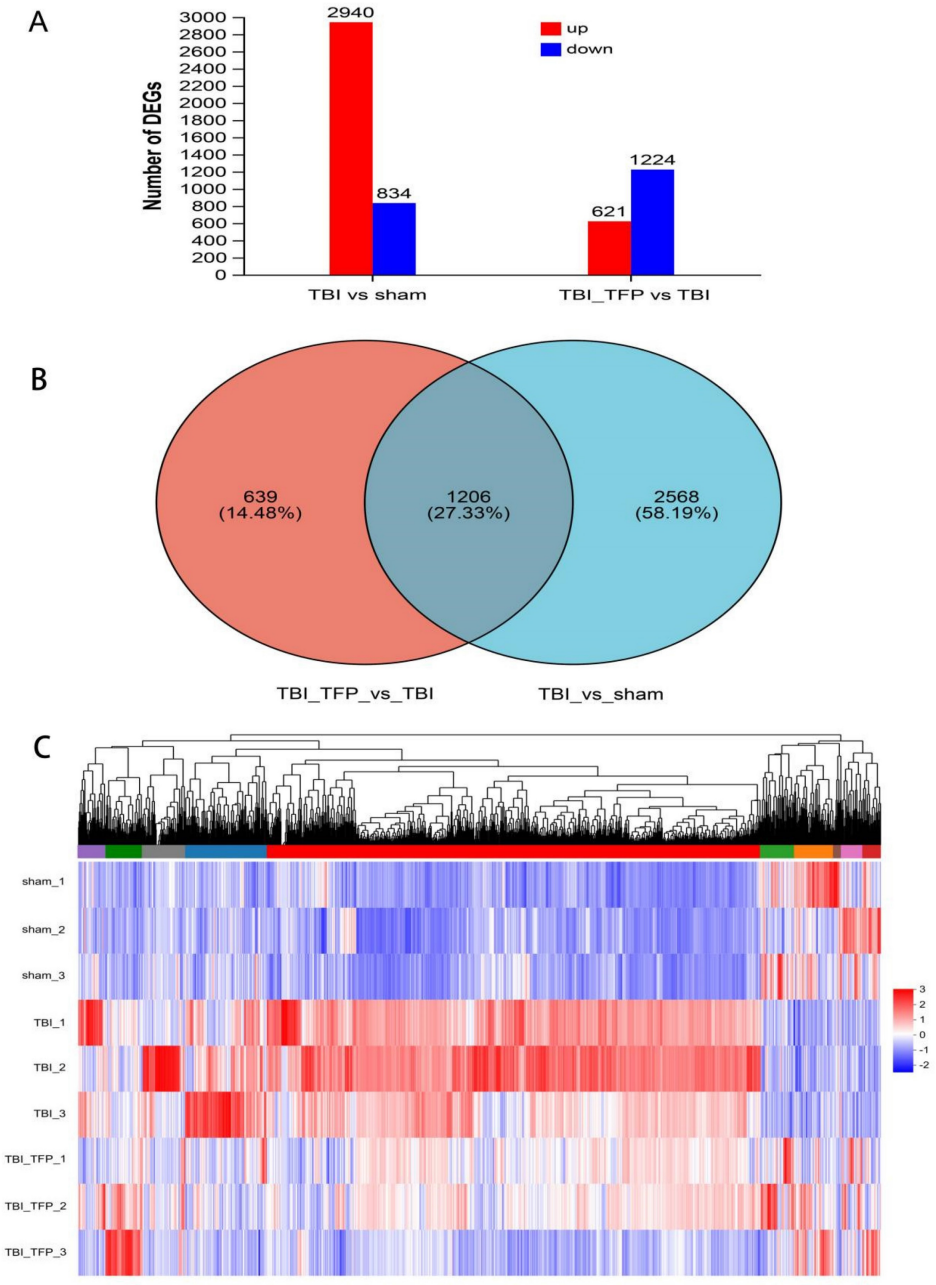
RNA-seq analysis in the Sham, TBI, and TBI+TFP groups. A: Histograms of DEGs between different groups; B: Venny analysis between DEGs in TBI vs Sham and DEGs in TBI+TFP vs TBI; C: Cluster analysis of three groups (Sham, TBI, and TBI+TFP groups).

**Figure 4:**
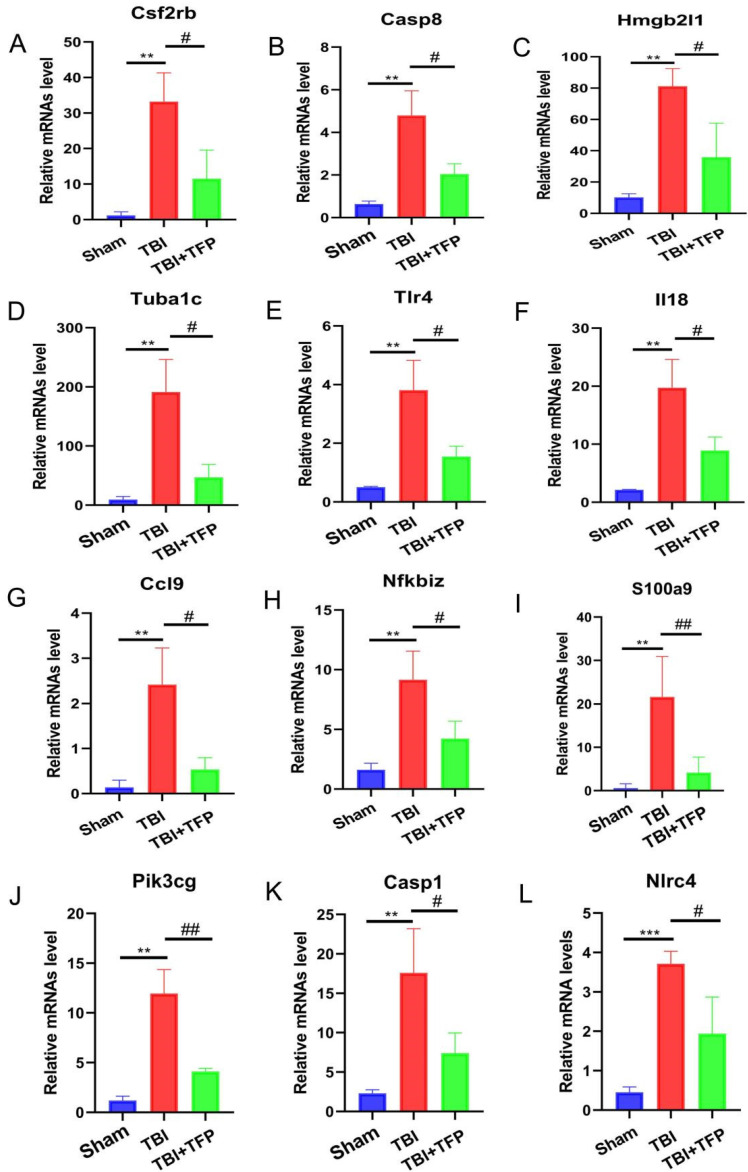
The mRNA expression levels of different apoptosis and inflammation genes. ***P < 0.001, **P < 0.01, *P < 0.05 was the comparison between the TBI and Sham groups, ^##^P < 0.01, ^#^P < 0.05 was the comparison between the TBI+TFP and TBI groups (n = 3 in each group).

**Figure 5:**
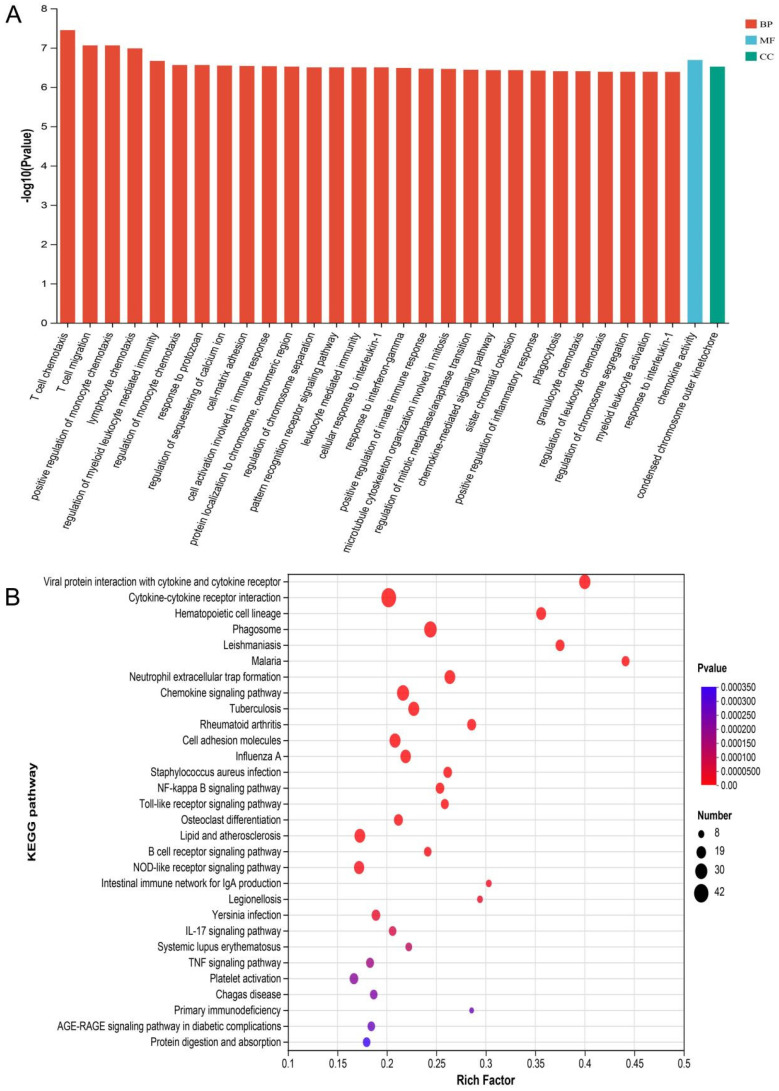
GO and KEGG analysis of DE mRNA. A: The top 30 GO terms of common differentially expressed mRNA in each group (Sham, TBI, and TBI+TFP groups). BP: biological process; CC: cellular component; MF: molecular function; B: The top 30 KEGG enrichment of common differentially expressed mRNA in each group (Sham, TBI, and TBI+TFP groups).

**Figure 6:**
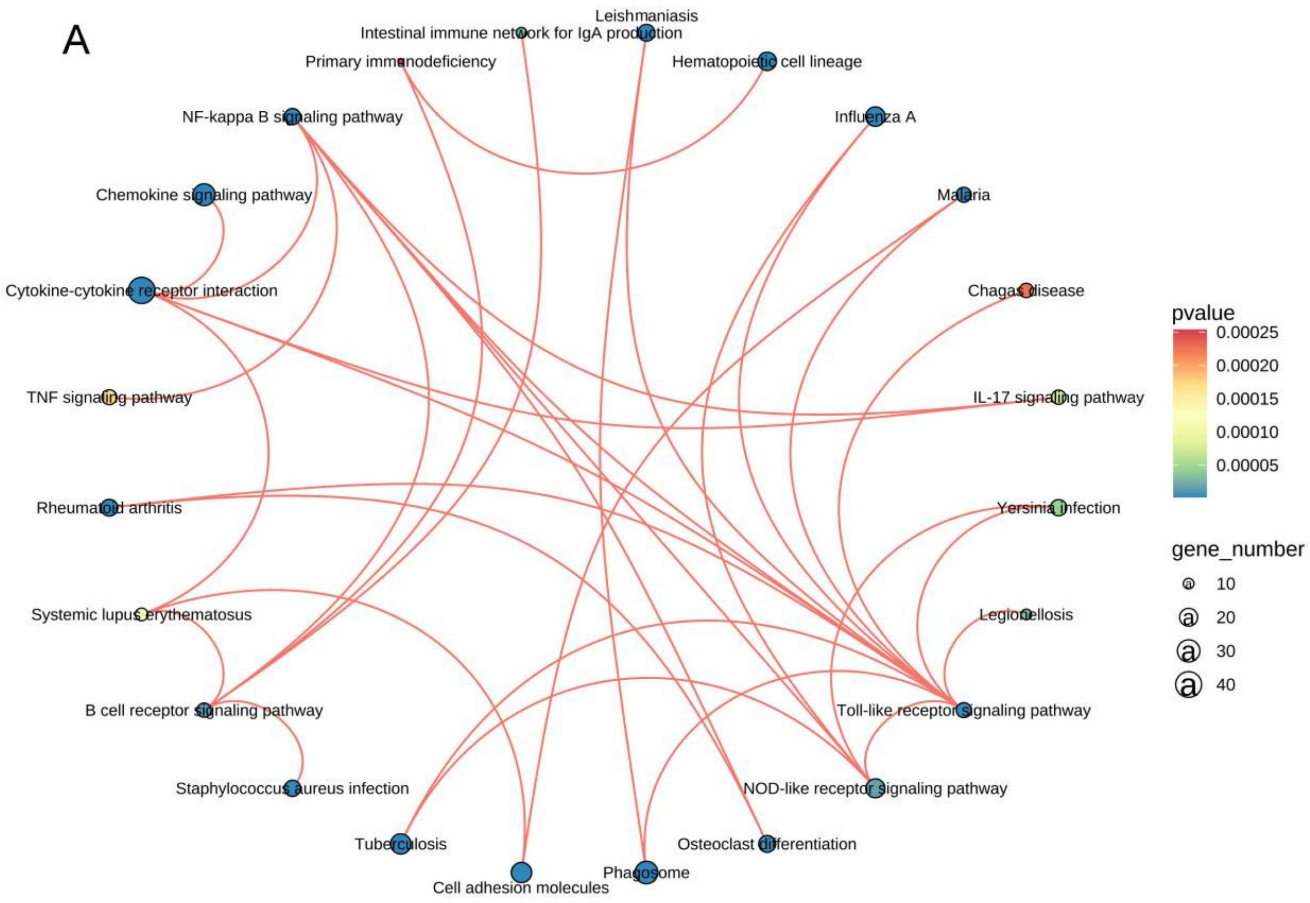
Network diagram of the top 30 KEGG enrichment pathways, 24 of these pathways were related to each other.

The mRNA for some genes were expressed differently among the Sham, TBI, and TBI+TFP groups (Figure 3A). However, TFP reversed the dysregulated gene expression (Figure 3B). Heat maps showing abnormal gene expression after TBI and abnormal gene expression after TBI reversal by TFP (Figure 3C). TFP reverses these abnormal regulatory genes involved in apoptosis and inflammation, as shown in the Figure 4, Table S1 and S2. GO and KEGG respectively counted the top 30 enrichment pathways and found that inflammatory signaling pathways were widely enriched (Figures 5A and 5B). The inflammatory pathways that are enriched by GO are these: chemokine-mediated signaling pathway, positive regulation of inflammatory response, regulation of leukocyte chemotaxis, cellular response to interleukin-1, and chemokine activity. The following inflammatory signaling pathways are enriched by KEGG, including cytokine-cytokine receptor interaction, chemokine signaling, NF-kappa B signaling, toll-like receptor signaling, NOD-like receptor signaling, IL-17 signaling, and TNF signaling pathways. A network interaction map of the top 30 KEGG enriched signaling pathways were constructed, we found that 24 of these pathways were related to each other (Figure 6A). Generally, these inflammatory signaling pathways are closely related to immune regulatory signaling pathways.

## 4. Discussion

In this study, we found that the relative immunofluorescence area and the relative immunofluorescence intensity of AQP4 around brain cells (astrocyte endfeet) increased significantly after TBI, but TFP treatment reversed these phenomena. The results showed that TFP inhibited AQP4 accumulation on the surface of brain cells (astrocyte endfeet). Meanwhile, the fluorescence area and content of the tunel reduced significantly after TFP treatment. Additionally, TFP alleviated brain edema, and reduced brain defect area and nervous system severity score (mNSS) improved. Genome-wide RNA-seq analysis of the cerebral cortex revealed that trifluoperazine reversed the abnormal expression of many genes regulating apoptosis and inflammation induced by TBI. The GO and KEGG enrichment analysis revealed that inflammatory signaling pathways were highly enriched. These results suggest that TFP can alleviate brain edema after TBI by preventing the accumulation of AQP4 on the surface of brain cells. This alleviates apoptosis and inflammation induced by TBI, thus improving nerve function after TBI.

Cerebral edema, a common feature of brain injury, can increase the intracranial pressure and cerebral ischemia, associated with poor prognosis. Currently, the treatment of cerebral edema mainly focuses on reducing intracranial pressure. However, no treatment that targets the pathways involved in edema onset and progression has been found 24. Previous studies have shown that silencing the AQP4 gene could reduce edema caused by TBI 25. However, so far, no specific therapeutic agent exists against the water flux caused by AQP4 25.

Trifluoperazine (TFP) is a clinical antipsychotic drug 16. TFP has been found to reduce central nervous system edema by affecting the subcellular localization of AQP4 in astrocytes 16. In this experiment immunofluorescence analysis revealed that TFP treatment downregulated the expression of AQP4 around cerebrovascular endothelial cells (Figures 1B and 1C). However, there was no significant difference in AQP4 expression around astrocytes and microglia after TFP treatment, as shown in Figure 1D-G, which might be related to the difference in AQP4 abundance around brain cells of each group. Because AQP4 is mainly distributed in the blood-brain barrier, in particular, the most abundant distribution is in the endfeet of the astrocyte 14. AQP4 in the astrocyte endfeet is mainly located around the blood vessels in the brain, and regulate the water balance between astrocytes and the blood-brain barrier 14, 26, 27. Therefore, AQP4 around cerebrovascular endothelial cells in this study reflects the presence of AQP4 in the endfeet of astrocytes. These results indicate that TFP can reduce AQP4 expression in astrocyte enffeet. Statistical results of brain edema in our experiment also support this phenomenon. The degree of brain edema in rats reduced after TFP treatment (Figure 2F), consistent with previous findings 16.

AQP4 is an important water transporter. It has been confirmed that TBI can increase the expression of AQP4 in the brain 15. In this study, the increase of the AQP4 water channel around brain cells (astrocyte endfeet) after TBI allows water into the cerebral astrocytes through the blood-brain barrier, causing brain cell edema. In contrast, TFP prevents this process, thus reducing brain edema. Other studies have shown that AQP4 participates in the development and alleviation of edema after brain injury. Early inhibition of water channels can prevent edema development after brain injury. However, in the later stage of the disease development, AQP4 can clear water from the brain into blood vessels. These provides evidence for early intervention of AQP4 to reduce brain edema. The dual and complex functions of AQP4 make it an excellent target for TBI treatment 21. In this study, the total fluorescence area and fluorescence intensity of the tunel significantly reduced after TFP treatment (Figures 2B and 2C). TFP treatment reduce the brain defect area and modified neurological severity score (mNSS) (Figures 2E and 2G), indicating that TFP can alleviates brain edema after TBI and reduce apoptosis of brain cells by preventing AQP4 aggregation on the surface of the brain cells, improving neurological function.

Previous studies on AQP4 knockout mice have shown that AQP4 participates in brain water balance, nerve excitation, glial scar, neuroinflammation, development of neurodegenerative diseases and neuropsychiatric disorders 28. The expression of AQP4 is influenced by various molecular mechanisms, including neuronal high mobility group box1, FOXO3a, vascular endothelial growth factor, hypoxia-inducing factor-1α (HIF-1α), sirtuin2, NF-kB, Malat1, nerve growth factor, and angiotensin II receptor type1, etc. 23. However, so far, the specific genes and signaling pathways involved in the regulation of AQP4 by TFP remain unclear. Whole genome RNA-seq analysis of the cerebral cortex tissues identified 3774 DEGs between the TFP treatment and the Sham group. Of these, the expression of 2940 genes were significantly up-regulated, whereas that of 834 was significantly down-regulated. A total of 1845 DEGs were also identified between the TBI+TFP and the TBI groups. Of these, 621 genes were overexpressed and 1224 genes were under expressed (Figure 3A). Further analysis showed that TFP reversed some of the abnormally expressed genes after TBI (Figures 3B and 3C). In our study, some of the apoptotic genes reversed by TFP are Csf2rb, Hmgb2l1, Casp8, and Tuba1c, etc. (Figures 4A-D, Table S1), and calmodulin-related signaling pathways (regulation of sequestering of calcium ion) were enriched by GO (Figure 5A). Well-known strong inhibition of calmodulin activity by TFP 29, 30. TFP has previously been reported to inhibit apoptosis, TFP may prevent doxorubicin-induced mitochondrial permeability transition pore (mPTP) opening and myocardial cell apoptosis through calmodulin-dependent mechanisms 31. Calcium overload and the subsequently activated calcium-calmodulin cascade can trigger the opening of mPTP, resulting in cardiomyocyte death 32-34. In the study of brain cells, it has been found that one of the mechanisms causing apoptosis of brain cells may be due to mitochondrial Ca^2+^ overload, and involves the opening of mPTP 35. In our study, we found that the regulaion of sequestering of calcium ion signaling pathway is enriched by GO, After TFP treatment, many apoptotic genes were reversed, and the accumulation of AQP4 on the surface of brain cells was reduced, and the tunel signal reflecting cell apoptosis was reduced. TFP, as a calmodulin blocker, may prevent cell cell apoptosis by blocking mPTP opening and reducing AQP4 accumulation on the surface of brain cells.

TFP treatment abrogated the abnormal expression of inflammation-related genes (Tlr4, il18, Ccl9, Nfkbiz, S100A9, Pik3cg, Casp1, and Nlrc4 etc.) (Figures 4E-L, Table S2). The GO and KEGG enrichment analysis revealed the signaling pathways to which TFB exerts its effect. In the top 30 most important pathways, signaling pathways associated with inflammation include positive regulation of inflammatory response, regulation of leukocyte chemotaxis, cellular response to interleukin-1, chemokine signaling pathway, cytokine-cytokine receptor interaction, NF-kappa B signaling pathway, NOD-like receptor signaling pathway, IL-17 signaling pathway and TNF signaling pathway are shown in Figures 5A and 5B. Studies have shown that AQP4 regulates inflammation in the brain 14. High expression of AQP4 mRNA has been found in many models of brain inflammatory diseases 23, including bacterial meningitis 36. Intraventricular injection of lipopolysaccharide (LPS) also induces overexpression of AQP4 37. Studies have found that both NF-kappa B signaling pathway and AQP4 are involved in the regulation of cerebral edema after TBI 38. As a calmodulin blocker, TFP has regulatory relationship with NF-kappa B signaling pathway 39. Previous in vivo results have shown that TFP has a strong inhibitory effect on calcium/calmodulin signaling, myocardial NF-kappa B activation can occur downstream of the calcium-calmodulin signaling 40, 41. Studies in mice have found that doxorubicin induced the expression of myocardial NF-kappa B-p65 which was markedly inhibited by TFP, suggesting that cardio protection conferred by TFP involved, at least in part, suppression of NF-kappa B 31. Regulation of sequestering of calcium ion and NF-kappa B signaling pathway was also observed in our study (Figure 5B). The gene Nf-kbiz (Figures 4I), which reflect inflammation was reversed by TFP. Trifluoperazine-mediated calmodulin antagonism affects AQP4 accumulation on the surface of brain cells and inhibits NF-kappa B gene expression, which may at least partly explain the protective effect of TFP against TBI.

In this study, the network interaction diagram of the top 30 signal pathways enriched by KEGG (Figure 6A) further indicates that these signal pathways are closely related. Interestingly, we found a complex interaction network among inflammation and immune response pathways, demonstrating the mutual regulatory relationship (Figures 5 and 6). It may be because the surge of immune cells and increase in inflammatory mediators at the lesion site develops a self-propagating amplification of a pro-inflammatory environment, furthering the exacerbation of secondary injury 17. TFP acts on AQP4 to reduce inflammation and apoptosis, which may be the result of the co-regulation of these signaling pathways. Studies have shown that AQP4 could be a target for treating neuromyelitis optical spectrum disorder (NMOSD) and other inflammatory autoimmune diseases 42. Brain-derived central nervous system (CNS) reactive T cells open the blood-brain barrier, allowing entry of human AQP4-IgG to the cerebral blood-brain barrier 43-46. AQP4-IgG binds to astrocytes to induce interleukin-6 production, which potentially decreases blood-brain barrier function, increases chemokine production and leukocyte transport, and forms feed-forward pathological loop 47. These results suggest that TFP may reduce AQP4 in brain cells indicating aggregation and modulates inflammation and immune responses. Inhibition of AQP4, regulation of inflammation and suppression of immune response may be the three treatment methods for cerebral edema after TBI. These signaling pathways and networks could be targeted in future research and mechanism of TBI pathogenesis.

In conclusion, by preventing AQP4 from accumulating on the surface of brain cells, TFP can improve the edema of brain cells after TBI, reduce the apoptosis and inflammatory reaction of brain cells induced by TBI, and thus promote the recovery of nerve function in rats after TBI. TBI pathogenesis is mainly mediated by apoptosis, inflammatory and immune response. Thus, these pathways could be targeted for TFP treatment. Generally, the findings of this study suggested that TFP could be used for TBI treatment.

## Supplementary Material

Supplementary table 1.

Supplementary table 2.
